# Disconnectome associated with progressive white matter hyperintensities in aging: a virtual lesion study

**DOI:** 10.3389/fnagi.2023.1237198

**Published:** 2023-08-31

**Authors:** Meng Li, Mohamad Habes, Hans Grabe, Yan Kang, Shouliang Qi, John A. Detre

**Affiliations:** ^1^College of Medicine and Biological Information Engineering, Northeastern University, Shenyang, China; ^2^Department of Neurology, University of Pennsylvania, Philadelphia, PA, United States; ^3^Biggs Alzheimer’s Institute, University of Texas San Antonio, San Antonio, TX, United States; ^4^Department of Psychiatry and Psychotherapy, University of Greifswald, Stralsund, Germany; ^5^College of Health Science and Environmental Engineering, Shenzhen Technology University, Shenzhen, China; ^6^Key Laboratory of Intelligent Computing in Medical Image, Ministry of Education, Northeastern University, Shenyang, China

**Keywords:** white matter hyperintensities, disconnectome, aging, brain network, diffusion tensor imaging, magnetic resonance imaging

## Abstract

**Objective:**

White matter hyperintensities (WMH) are commonly seen on T2-weighted magnetic resonance imaging (MRI) in older adults and are associated with an increased risk of cognitive decline and dementia. This study aims to estimate changes in the structural connectome due to age-related WMH by using a virtual lesion approach.

**Methods:**

High-quality diffusion-weighted imaging data of 30 healthy subjects were obtained from the Human Connectome Project (HCP) database. Diffusion tractography using q-space diffeomorphic reconstruction (QSDR) and whole brain fiber tracking with 10^7^ seed points was conducted using diffusion spectrum imaging studio and the brainnetome atlas was used to parcellate a total of 246 cortical and subcortical nodes. Previously published WMH frequency maps across age ranges (50’s, 60’s, 70’s, and 80’s) were used to generate virtual lesion masks for each decade at three lesion frequency thresholds, and these virtual lesion masks were applied as regions of avoidance (ROA) in fiber tracking to estimate connectivity changes. Connections showing significant differences in fiber density with and without ROA were identified using paired tests with False Discovery Rate (FDR) correction.

**Results:**

Disconnections appeared first from the striatum to middle frontal gyrus (MFG) in the 50’s, then from the thalamus to MFG in the 60’s and extending to the superior frontal gyrus in the 70’s, and ultimately including much more widespread cortical and hippocampal nodes in the 80’s.

**Conclusion:**

Changes in the structural disconnectome due to age-related WMH can be estimated using the virtual lesion approach. The observed disconnections may contribute to the cognitive and sensorimotor deficits seen in aging.

## 1. Introduction

White matter hyperintensities (WMH) are commonly seen in older subjects on fluid-attenuated inversion recovery (FLAIR) magnetic resonance imaging (MRI) and increase with age from small lesions to large confluent lesions (Jorgensen et al., 2018). WMH are thought to primarily reflect small vessel ischemic disease (Debette and Markus, 2010; Prins and Scheltens, 2015; Alber et al., 2019) while increased WMH are also associated with Alzheimer’s disease (AD) neuropathology (Debette and Markus, 2010; Brickman et al., 2015; Prins and Scheltens, 2015; Alber et al., 2019; Liang et al., 2023). Although the main risk factor for WMH is age (Garnier-Crussard et al., 2020; Guevarra et al., 2020), vascular risk factors such as smoking and diabetes mellitus contribute to increased WMH volumes (Debette and Markus, 2010; Verdelho et al., 2010; Prins and Scheltens, 2015; Alber et al., 2019). WMH are associated with cognitive decline, particularly in the domains of information-processing speed and executive function (De Groot et al., 2002; Garde et al., 2005; Prins et al., 2005). The location of WMH is another factor in their clinical impact (Alber et al., 2019). WMH associated with cerebral small vessel disease are most commonly located in periventricular and deep subcortical regions (Debette and Markus, 2010; Prins and Scheltens, 2015; Habes et al., 2018; Phuah et al., 2022), with periventricular versus deep WMH showing differential effects on both cognition (van Dijk et al., 2008) and regional cortical atrophy (Mayer et al., 2021).

One key mechanism by which WMH are thought to affect cognition is through the disconnection of associated gray matter regions (Jaywant et al., 2020). Structural connectivity in the human brain can be inferred using diffusion tractography, and several studies have used diffusion tensor imaging (DTI) data to show that alterations in overall brain network connectivity are associated with cognitive deficits in cohorts of older subjects (Tuladhar et al., 2016; Wiseman et al., 2018). Associations between structural connectivity changes and cognition have also been demonstrated across individual connections (Langen et al., 2018). That study examined both connectivity and disconnections, finding that the latter had greater explanatory power for cognitive deficits. Recently, a study found that the altered structural network due to WMH in AD may predict the progress of the disease (Liang et al., 2023).

Diffusion tensor imaging is more sensitive to changes in white matter due to cerebral small vessel disease than conventional MRI (Lockhart et al., 2012; Zhang et al., 2013), but diffusion tractography in the presence of WMH is also prone to artifacts (Reginold et al., 2016, 2018). An alternative strategy to avoid this potential confound is a virtual lesion approach, which uses WMH masks derived from older subjects as regions of avoidance in diffusion tractography data from healthy subjects to estimate connectivity changes (Kuceyeski et al., 2013). From our previously published work, a virtual lesion approach was used to estimate connectivity changes due to periventricular WMH (Li et al., 2021) simulated by the CBF map generated from the perfusion MRI (Dolui et al., 2019; Li et al., 2021). In another recent study, we also confirmed that the use of young healthy subjects for virtual lesion tractography was preferable to older subjects due to their higher streamline counts and the absence of WMH lesions (Taghvaei et al., 2023).

Because age is the main risk factor for WMH, most cohort studies adjust for age in assessing brain-behavior correlations based on structural integrity. Here we instead sought to characterize the effect of age on the magnitude and spatial distribution of WMH-associated disconnections. To accomplish this, we used WMH frequency maps across different age ranges (50’s, 60’s, 70’s, and 80’s) derived from a previously published large sample (*n* = 2,698) (Habes et al., 2016) and selected a series of frequency thresholds for simulating progressive WMH with increasing volume and spatial extent. These thresholded WMH frequency maps were applied as regions of avoidance (ROA) for diffusion tractography in healthy subjects from the Human Connectome Project. By comparing the structural connectome with and without virtual lesions, we determined significant disconnections associated with progressive WMH in aging.

## 2. Materials and methods

### 2.1. Healthy subject diffusion data from the human connectome project

High-quality MRI data for 30 healthy subjects in the age range of 22 and 35 years (15 Females, 5 subjects aged between 22 and 25 years, 17 subjects aged between 26 and 30 years, and 8 subjects aged between 31 and 35 years) from the Human Connectome Project (HCP) database were included (Van Essen et al., 2013). Healthy young subjects in the HCP were chosen to represent healthy adults beyond the age of major neurodevelopmental changes and before the onset of neurodegenerative changes (Van Essen et al., 2012, 2013). The DTI data size of *N* = 30 in this study is also the same as in our prior work (Li et al., 2021). The Institutional Review Board (IRB) of Washington University in Saint Louis approved the acquisition protocol to acquire the subjects’ MRI images. HCP DTI data were scanned on a customized 3T Siemens Skyra scanner utilizing the Stejskal-Tanner pulsed gradient (i.e., monopolar) scheme, within a single-shot 2D spin echo multiband EPI acquisition. The followings are the parameters used: TR = 5,520 ms, TE = 89.5 ms, flip angle = 78°, echo spacing = 0.78 ms, slices = 111, isotropic voxels = 1.25 mm, matrix size = 168 × 144, FOV = 210 × 180 mm^2^, directions = 90. Sampling in q-space includes three shells at *b* = 1,000, 2,000, and 3,000 s/mm^2^ with six *b* = 0 images (Sotiropoulos et al., 2013). The HCP DTI data we used are freely available at the Human Connectome Projects website:,^1^ and the scripts used in the study are available from the corresponding author upon request.

### 2.2. Overview of the data analysis procedure

**FIGURE 1:**
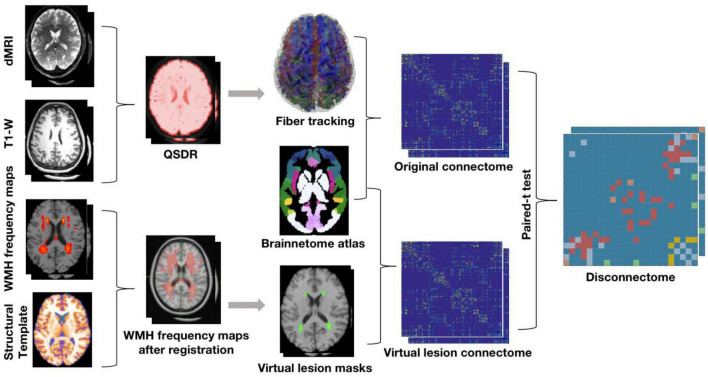
The pipeline of obtaining the DTI structural disconnectome.

The data analysis strategy is illustrated in Figure 1. High angular resolution diffusion tractography and T1-weighted structural MRI data from *N* = 30 healthy young subjects were selected from the HCP database. The orientation density function was estimated by using the q-space diffeomorphic reconstruction (QSDR) method and whole brain fiber tracking was conducted using diffusion spectrum imaging (DSI) studio with 10^7^ seed points. The brainnetome atlas (see section “2.4. “White matter fiber tractography and structural connectivity network construction” below) was used to parcellate cortical and subcortical nodes to define the structural connectome in the *N* = 30 HCP subjects. WMH frequency maps across different age ranges derived from a previously published large sample (see section “2.3. “Virtual lesion masks from WMH frequency maps” below) were registered to MNI space and used as ROA for virtual lesion tractography in the *N* = 30 HCP subjects. Finally, disconnections with significant differences (FDR correction at *p* < 0.01) before and after the application of virtual lesion were determined using paired *t*-tests between the original connectome and virtual lesion connectome.

### 2.3. Virtual lesion masks from WMH frequency maps

White matter hyperintensities frequency maps were derived from a large population-based sample (*n* = 2,698) enrolled in the Study of Health in Pomerania (SHIP), which included MRI (Habes et al., 2016). WMH frequency maps were generated for each age decade (40’s *n* = 538, 50’s *n* = 668, 60’s *n* = 610, 70’s *n* = 470, and 80’s *n* = 106). The WMH frequency maps were defined originally in Jackob space (Habes et al., 2016) and were transformed into Montreal Neurological Institute (MNI) space by firstly co-registering to the structural MRI, then normalizing the structural MRI to MNI space, and finally inverting the two transformation matrices to obtain the WHM frequency maps in MNI space. Virtual lesion masks were generated using frequency thresholds of 0.02, 0.10, and 0.20 at four age groups (50’s, 60’s, 70’s, and 80’s). We selected these low, medium, and high thresholds for generating WMH lesion masks from WMH frequency maps to capture the range of possible disconnections at each decade.

### 2.4. White matter fiber tractography and structural connectivity network construction

Nodes for connectivity analyses were generated using the brainnetome atlas which parcellates the brain into 210 cortical and 36 subcortical subregions with both anatomical and functional network relevance (Fan et al., 2016).

DSI Studio^2^ was used to reconstruct the diffusion data and calculate the connections (edges) in the structural connectivity network. Firstly, 90 sampling directions and six b0 image sets were loaded, and masks for filtering out the background region and increasing the reconstruction efficacy were created. Next, we selected the QSDR method to conduct the tractography in the MNI space. For deterministic whole brain fiber tracking, the parameters of 10^7^ seed points and fiber length (from 20 to 400 mm) were used to calculate the structural connectome (Power et al., 2011). To calculate the density-weighted structural connectivity matrix, we used the sum of the volume of each pair of subregions to divide the number of tracts connecting each pair of subregions and the sparsity of the density-weighted structural connectivity matrix was set at 0.9 (Qi et al., 2015, 2016; Roberts et al., 2017), which means that 90% of the elements are zero in the matrix. Connectivity matrices were calculated with and without virtual lesion masks for the *N* = 30 healthy subjects. In DSI Studio, virtual lesion masks are considered as regions of avoidance and any streamlines passing through these regions are deleted.

### 2.5. Virtual lesion disconnectome

To detect changes in the structural disconnectome caused by the different frequency thresholds of WMH in each age-decade group (from the 50’s to the 80’s), we conducted paired *t*-tests between the original connectome and the virtual lesion connectome at each frequency threshold. We considered disconnections surviving FDR correction at *p* < 0.01 as significant.

### 2.6. Number and proportion of disconnected streamlines at significant disconnections

To further characterize the extent of white matter streamlines affected by WMH, we calculated the number and proportion of disconnected streamlines at each significant disconnection. The number of streamlines in each brain region of the original connectome and that of the virtual lesion connectome across the various WMH lesion masks were counted for the *N* = 30 subjects and the proportion of disconnected streamlines was obtained by dividing the decreased number of streamlines with the lesion mask by the original streamline number in every brain region.

## 3. Results

### 3.1. WMH masks and volume with aging

**FIGURE 2:**
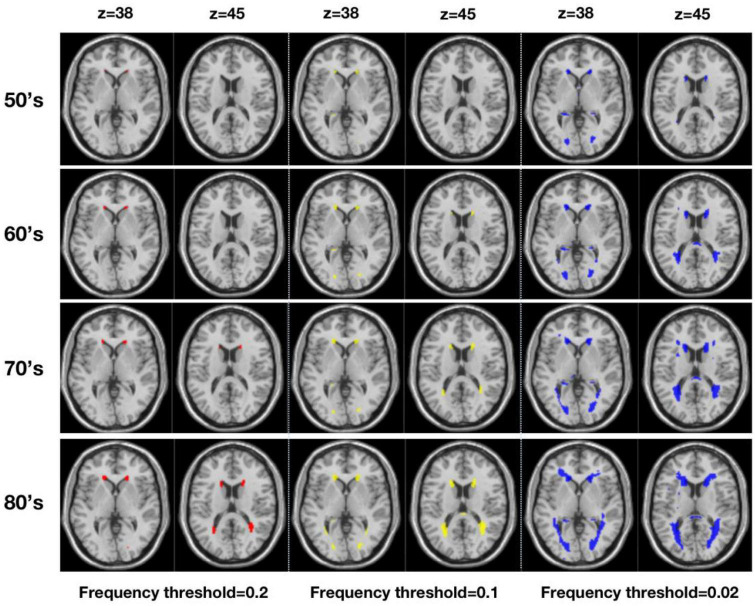
Virtual lesion masks generated from the white matter hyperintensities maps derived from a large population-based sample (*n* = 2,698) and registered to MNI standard space (Three frequency thresholds of 0.20 (red), 0.10 (yellow), and 0.02 (blue) are applied for four age ranges from the 50’s to the 80’s).

**FIGURE 3:**
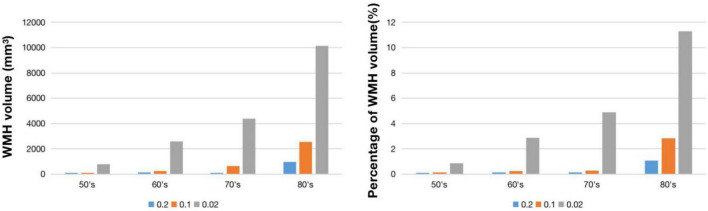
WMH volume **(left)** and percentage of WMH volume **(right)** under different frequency thresholds across age ranges.

Figure 2 shows WMH lesion masks for subjects in their 50’s, 60’s, 70’s, and 80’s at three frequency thresholds: 0.02, 0.10, and 0.20. Subtle WMH lesions appear in the frontal lobe and subcortex in the 50’s, and both enlarge and extend in the periventricular regions with increasing age and decreasing lesion frequency threshold. The volume represented by these WMH masks varies from 20 to 10,138 mm^3^, and the corresponding percentage is from 0.02 to 11.3% (Figure 3). For the frequency thresholds of 0.2 and 0.1, WMH volume increases exponentially with age. For example, under frequency threshold 0.1, WMH volume increases by 2.22 (from the 50’s to the 60’s), 2.39 (from the 60’s to the 70’s), and 3.99 times (from the 70’s to the 80’s). While under frequency threshold 0.02, there are fluctuations in the growth rate of WMH volume with the increasement of 3.36 (from the 50’s to the 60’s), 1.70 (from the 60’s to the 70’s), and 2.31 times (from the 70’s to the 80’s), respectively.

### 3.2. Virtual lesion disconnectome with aging

**FIGURE 4:**
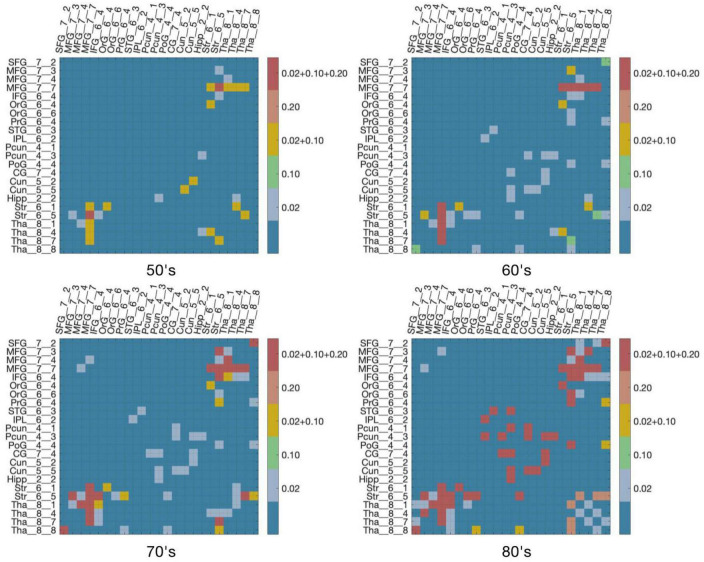
DTI disconnectome derived by virtual lesion masks through using thresholded WMH frequency map. The color scale shows the WMH frequency thresholds at which significant disconnections (*p* < 0.01, FDR correction) are observed. Parcels without connections are canceled in the matrix under the virtual lesion mask of frequency 0.2, age 80’s. The original estimated disconnection matrix can be found in Supplementary Figures 1–4.

**FIGURE 5:**
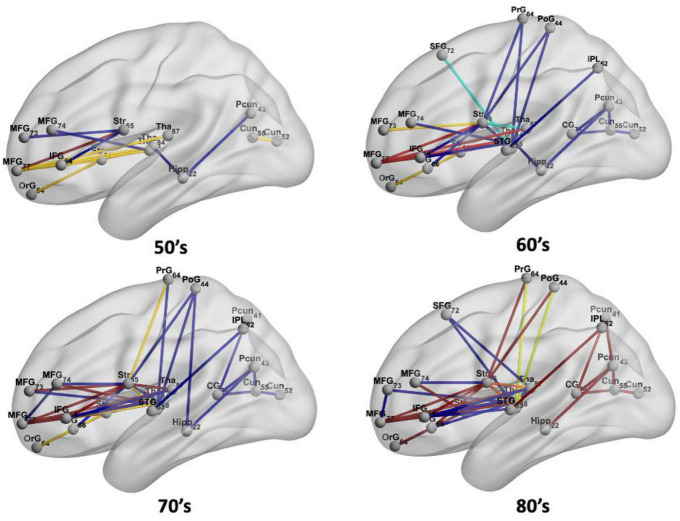
The spatial locations of disconnections at frequency thresholds including 0.02 (deep blue), 0.10 (light blue), 0.02 + 0.10 (yellow), 0.20 (orange), and 0.02 + 0.10 + 0.20 (red) in each age group.

**FIGURE 6:**
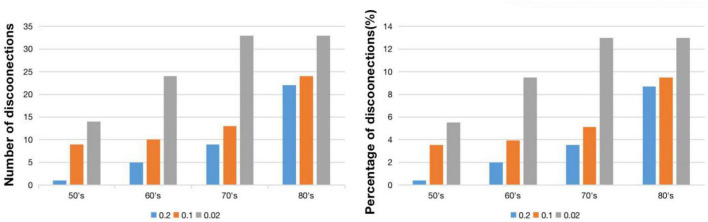
The number **(left)** and percentage **(right)** of the significant disconnections.

Figure 4 demonstrates virtual lesion disconnectome with estimated disconnections (*p* < 0.01, FDR correction) across 30 healthy HCP subjects at the four age groups color-coded for varying WMH mask thresholds. Figure 5 shows the spatial locations of disconnections at different frequency thresholds in each age group. Figure 6 shows the number and the percentage of significant disconnections.

In the 50’s, one (0.4%), nine (3.56%), and fourteen (5.53%) edges are disconnected under the frequency thresholds of 0.2, 0.1, and 0.02, respectively, and the disconnections are distributed in the middle frontal gyrus, inferior frontal gyrus, striatum and thalamus. The overlapping disconnection is between the striatum and middle frontal gyrus under all three frequency thresholds. In the 60’s, the proportion of disconnections is 1.98, 3.95, and 9.49% of edges under each frequency threshold, respectively across lesion frequency thresholds, and disconnections extend to appear in the superior frontal gyrus, superior temporal gyrus, inferior parietal lobe, hippocampus, postcentral gyrus, precuneus, cingulate gyrus and cuneus. Five overlapping disconnections are identified, involving brain regions connected with the striatum and middle frontal gyrus (also appearing in the 50’s) and with the thalamus. In the 70’s, the proportion of disconnections is 3.56, 5.14, and 13% of edges, respectively across lesion frequency thresholds, with the distributions being largely consistent with that found in the 60’s, but disconnections are also observed with superior frontal gyrus. In the 80’s, disconnections account for 8.7, 9.49, and 13% of edges, respectively across lesion frequency thresholds, and across almost all edges affected in earlier decades. Seventeen affected edges involve fourteen regions with the striatum, middle frontal gyrus, thalamus, superior frontal gyrus, inferior frontal gyrus, orbital gyrus, precentral gyrus, superior temporal gyrus, angular gyrus, precuneus, cuneus, postcentral gyrus, cingulate gyrus, hippocampus. Ten additional nodes are added to the disconnectome compared to the results in the 70’s.

### 3.3. Number and proportion of disconnected streamlines at significant disconnections with aging

**FIGURE 7:**
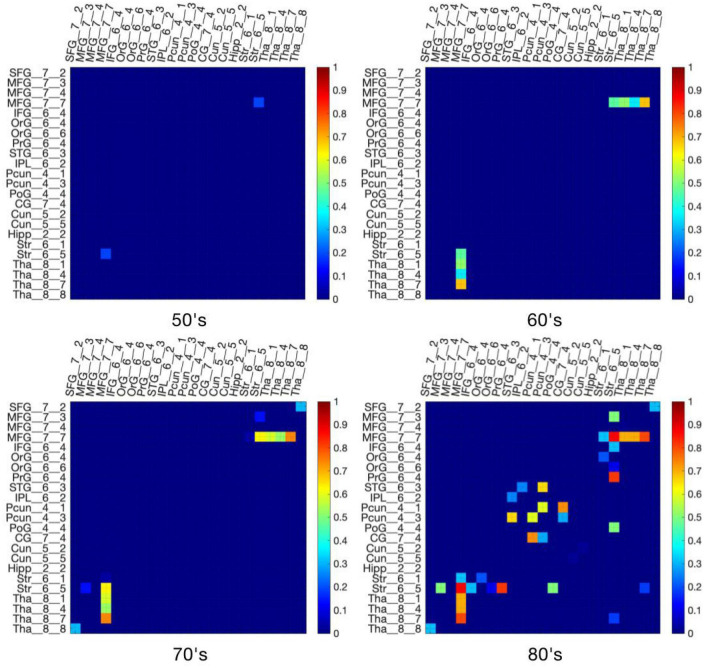
The proportion of lost streamlines due to virtual lesions was generated using a WMH frequency threshold of 0.2. The original proportion matrix can be found in Supplementary Figures 5–8.

**FIGURE 8:**
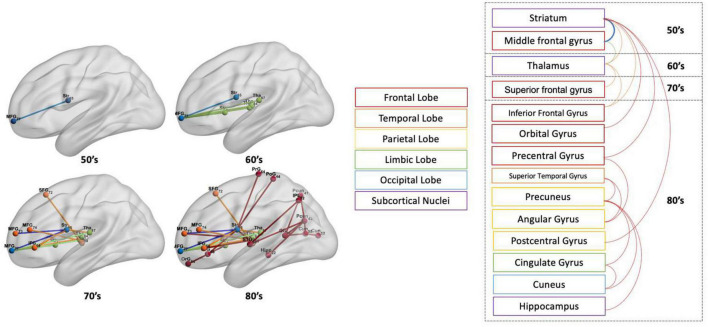
The spatial locations of disconnections **(left)** across ages including 50’s (blue), 60’s (blue and green), 70’s (blue, green, and orange), and 80’s (blue, green, orange, and red) at the frequency threshold of 0.2. On the **(right)** are the gyrus and lobes to which the corresponding disconnections are attached.

Figures 7, 8 show the structural disconnectome presented as a proportion of lost streamlines due to the virtual lesion masks. These results were generated using a lesion frequency of 0.2 since those results were the most stable across age ranges (see Figure 4). In Figure 7, the disconnection between the middle frontal gyrus_7_7 (lateral middle frontal gyrus) and striatum_6_5 (dorsal caudate) appears first in the 50’s with a proportion of disrupted streamlines of 19%. With increasing age, the proportion is 46% in the 60’s, 636% in the 70’s, and 872% in the 80’s. In the 60’s, five disconnections are observed. Among them, the disconnection between the middle frontal gyrus_7_7 and thalamus_8_7 (caudal temporal thalamus) shows the highest proportion of 68%. This proportion increases to 74% in the 70’s and 80% in the 80’s. In the 80’s, five disconnections have a proportion of disrupted streamlines higher than 60%. The new three disconnections are between the precentral gyrus_6_4 (trunk region) and striatum_6_5 (82%), between the precuneus_4_1(medial area of precuneus) and cingulate gyrus_7_4 (ventral posterior cingulate gyrus) (73%), between the precuneus_4_3 (dorsomedial parietooccipital sulcus) and superior temporal gyrus_6_3 (66%). Figure 8 shows the spatial locations of disconnections (left) across decades including 50’s (blue), 60’s (blue and green), 70’s (blue, green and orange), and 80’s (blue, green, orange and red) at the frequency threshold of 0.2. Detailed information on the lobe and regions with disconnections across different ages is also given (right). In both 50’s and 60’s the disconnection is between the frontal lobe (MFG) and the subcortex including the striatum and thalamus. In the 70’s, disconnections extend to the superior temporal gyrus and inferior frontal gyrus of the frontal lobe. In the 80’s, disconnections extend further to extensive the brain regions in the frontal lobe, subcortex, limbic system (e.g., hippocampus), partial lobe (e.g., postcentral gyrus, angular gyrus and precuneus) and occipital lobe (e.g., cuneus).

## 4. Discussion

We used a virtual lesion approach with population-based WMH frequency maps and normative diffusion tractography data to estimate changes in structural connectivity due to WMH with progressive aging. By performing virtual lesion tractography using normative diffusion MRI datasets, we estimated the age-dependent structural connectivity changes affected by progressive WMH lesions. Disconnections can be estimated directly using the HCP tractography template (Liang et al., 2023). However, to capture biological variability in tract locations and carry out statistical analysis to determine estimated connectivity changes, we applied a virtual lesion tractography approach in *N* = 30 subjects.

Subtle disconnections primarily involving subcortical to cortical connections seen in the 50’s increased in later decades and began to involve cortico-cortical connections in the 60’s, with more widespread disconnections of cortico-cortical connections in later decades. The disconnection profile we found is consistent with our prior study in which the CBF thresholded maps were used as virtual lesion masks to estimate the changes of connectivity through the simulation of the progressive periventricular white matter lesions (Li et al., 2021). In the current study, most of the WMH lesion burden in the WMH lesion frequency map used was also located in the periventricular region, appearing first as small ‘caps’ adjacent to the frontal horns of the lateral ventricles and later as caps near the occipital horns as well as in the thalamus and subcortical white matter. This is particularly the case for the lesion mask based on the highest lesion frequency threshold of 0.2 which yielded the most consistent results across the age groups. As expected, more widespread connectivity changes are observed using lower WMH thresholds, albeit with a somewhat less consistent progression across age ranges.

Although we did not measure cognition in this study, the spatial distribution of estimated disconnections we observed is consistent with the notion that age-related WMH changes underlie age-related changes in cognitive performance. For example, our finding that disconnection between the striatum and middle frontal gyrus appears early and increases progressively across older decades is consistent with the known role of these connections in age-associated decrements in memory and executive function (Buckner, 2004). Similarly, the observed disconnection between the thalamus and middle frontal gyrus also becomes more severe in older decades and is consistent with previous studies linking disrupted frontal-striatal-thalamocortical pathways with the decline of cognitive control (Jaywant et al., 2020). Disconnection of the precuneus to the cingulate gyrus (e.g., ventral posterior cingulate gyrus) is seen in the oldest decade. This region is the central node of the default mode network (DMN) (Raichle and Snyder, 2007), a key network underlying cognitive function in which functional connectivity is particularly compromised in aging (Sala-Llonch et al., 2015). Disconnection between the frontal lobe (e.g., precentral gyrus) and inferior parietal lobe (e.g., angular gyrus) seen in the oldest decade in our study are consistent with prior work demonstrating decreased global efficiency of the frontal-parietal network decreased in WMH subjects with cognitive impairment without dementia (Chen et al., 2019) and likely contributes to age-related decline in executive function (Jacobs et al., 2012). More widespread disconnections between the superior temporal gyrus and precuneus in the frontal lobe (e.g., dorsomedial parietooccipital sulcus) were seen in the 80’s. This, along with the disconnection between frontal and temporo-parietal cortex (e.g., precentral gyrus to angular gyrus, and angular gyrus to superior temporal gyrus), is consistent with a prior study linking analogous disconnections to deficits in the episodic memory performance (Lockhart et al., 2012). The main goal of this study was to demonstrate the utility of virtual lesion tractography for investigating the disconnectome due to age-associated WMH and to characterize the resulting age-related changes in the structural disconnectome using population-based WMH frequency maps. Future work using WMH masks from individual subjects for virtual lesion tractography may allow elucidating more detailed associations between the WMH disconnectome and cognitive performance.

The time course of estimated connectivity changes is also consistent with prior literature. A previous study showed that the dorsal periventricular WMH component was significantly associated with an Alzheimer’s disease (AD) polygenic risk score in individuals older than 65 years, and reflected a contribution of AD pathology to the development of WMH (Habes et al., 2018). In the current study, we found that disconnections increase exponentially in the 70’ and 80’s. Other studies also found the most significant alterations in functional connectivity in people aged 65 years and above with AD (Zonneveld et al., 2019; Edde et al., 2021). Estimated connectivity changes observed in the precuneus, ventral posterior cingulate gyrus, and hippocampus may well contribute to AD risk with aging (Kim et al., 2013; Yokoi et al., 2018; Van Etten et al., 2021).

There are several limitations in the present study. First, the sample size (*N* = 30) of the healthy subjects is modest. Second, some of the disconnected edges seen at lower mask thresholds did not persist across age groups, likely reflecting a sample size limitation of the WMH frequency maps and the target normative DTI data in accurately capturing random effects. It will be important to reproduce these findings using WMH frequency maps from additional cohorts, and with additional target healthy subject DTI data. Third, the extent of disconnection simulated using the virtual lesion approach may be overestimated since the ROA method models a complete disconnection. However, we note that none of the edges in the connectivity matrix were completely disconnected by lesion masks. We also note that the connectivity changes estimated using this approach only models direct connections between regions and does not consider indirect connections via intermediate nodes.

## 5. Conclusion

This study estimated the age-dependent structural disconnectome associated with progressive white matter hyperintensities during brain aging using a virtual lesion approach. The observed progression is from subcortical to cortical disconnections with increasing decades and the most consistent progression at the highest WMH lesion frequency threshold. Estimated connectivity changes increase dramatically above age 70, but even then most disconnections are partial. The observed disconnections might underlie some of the cognitive and sensorimotor deficits seen in aging and contribute to AD risk with aging.

## Data availability statement

Publicly available datasets were analyzed in this study. This data can be found here: https://www.humanconnectome.org.

## Ethics statement

The studies involving humans with DTI data from the HCP database were approved by Institutional Review Board (IRB) of Washington University in Saint Louis. The studies were conducted in accordance with the local legislation and institutional requirements.

## Author contributions

ML performed the experiments and analyzed the data along with MH, JD, and SQ. JD and MH conceived the study. ML wrote and revised the manuscript along with JD and SQ. JD, MH, YK, and SQ supervised the algorithm development. All authors read and approved the final manuscript.

## Footnotes

1
https://www.humanconnectome.org

2
http://dsi-studio.labsolver.org/
