# Supplementary material for: Disconnectome associated with progressive white matter hyperintensities in aging: a virtual lesion study
Source: Front Aging Neurosci. 2023 Aug 31;15:1237198. doi: 10.3389/fnagi.2023.1237198 (PMC10500060; doi:10.3389/fnagi.2023.1237198)

**Supplementary Figure 1.** DTI structural disconnectivity derived using thresholded WMH frequency maps as ROA for tractography. The connectivity matrix shows significantly reduced edges (p<0.01, FDR correction) across 30 healthy HCP subjects at the age of 50’s. The color scale shows the WMH frequency threshold at which significant disconnections were observed (three independent frequency thresholds of 0.02, 0.10, 0.20, and two superimposing frequency thresholds of 0.02+0.10 and 0.02+0.10+0.20).


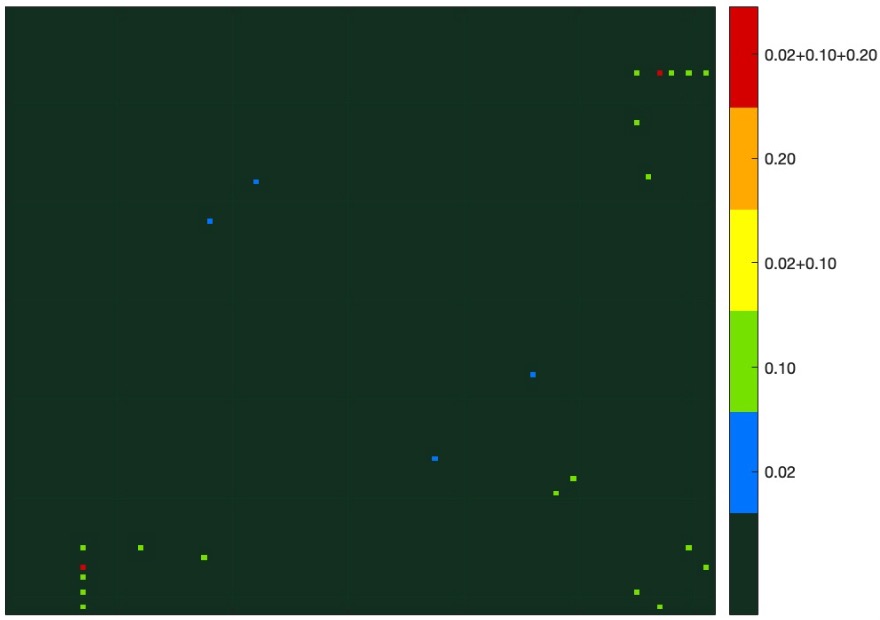


**Supplementary Figure 2.** DTI structural disconnectivity derived using thresholded WMH frequency maps as ROA for tractography. The connectivity matrix shows significantly reduced edges (p<0.01, FDR correction) across 30 healthy HCP subjects at the age of 60’s. The color scale shows the WMH frequency threshold at which significant disconnections were observed (three independent frequency thresholds of 0.02, 0.10, 0.20 and two superimposing frequency thresholds of 0.02+0.10 and 0.02+0.10+0.20).


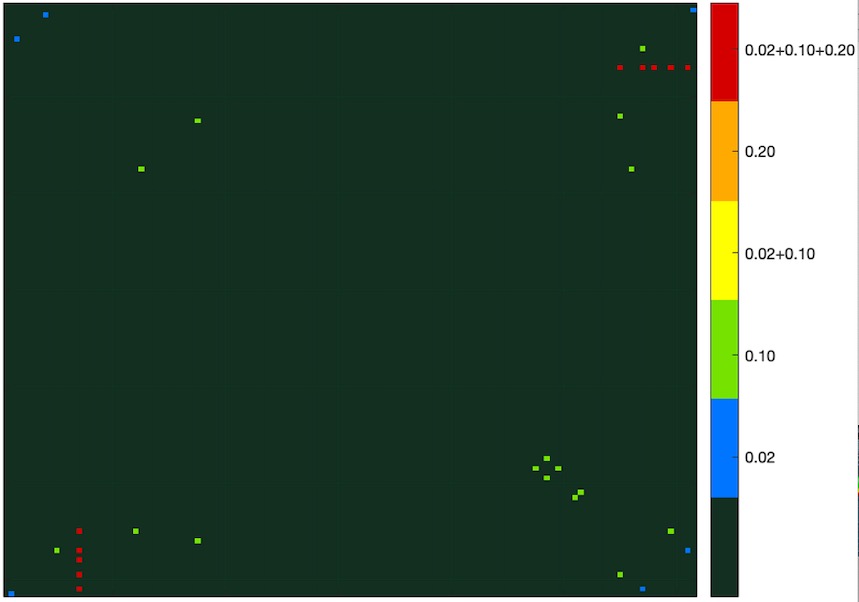


**Supplementary Figure 3.** DTI structural disconnectivity derived using thresholded WMH frequency maps as ROA for tractography. The connectivity matrix shows significantly reduced edges (p<0.01, FDR correction) across 30 healthy HCP subjects at the age of 70’s. The color scale shows the WMH frequency threshold at which significant disconnections were observed (three independent frequency thresholds of 0.02, 0.10, 0.20 and two superimposing frequency thresholds of 0.02+0.10 and 0.02+0.10+0.20).


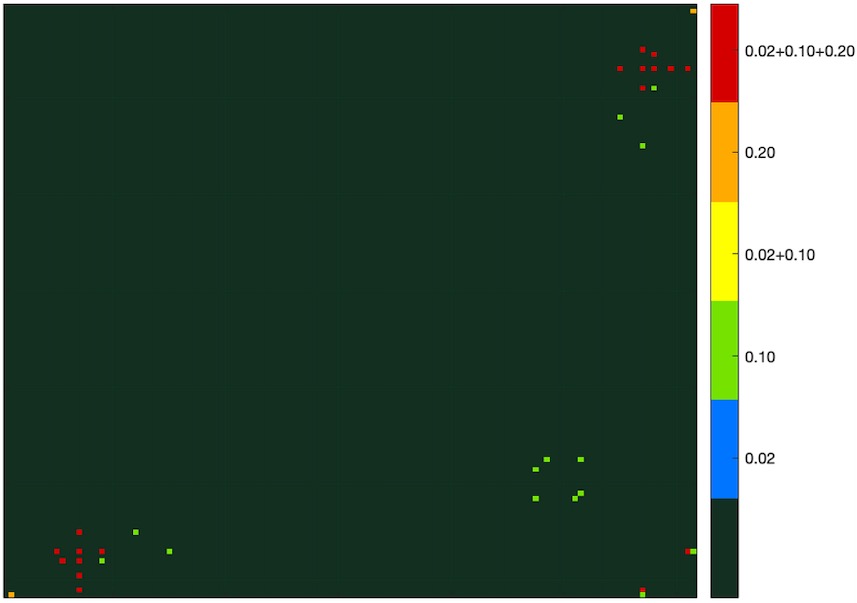


**Supplementary Figure 4.** DTI structural disconnectivity derived using thresholded WMH frequency maps as ROA for tractography. The connectivity matrix shows significantly reduced edges (p<0.01, FDR correction) across 30 healthy HCP subjects at the age of 80’s. The color scale shows the WMH frequency threshold at which significant disconnections were observed (three independent frequency thresholds of 0.02, 0.10, 0.20 and two superimposing frequency thresholds of 0.02+0.10 and 0.02+0.10+0.20).


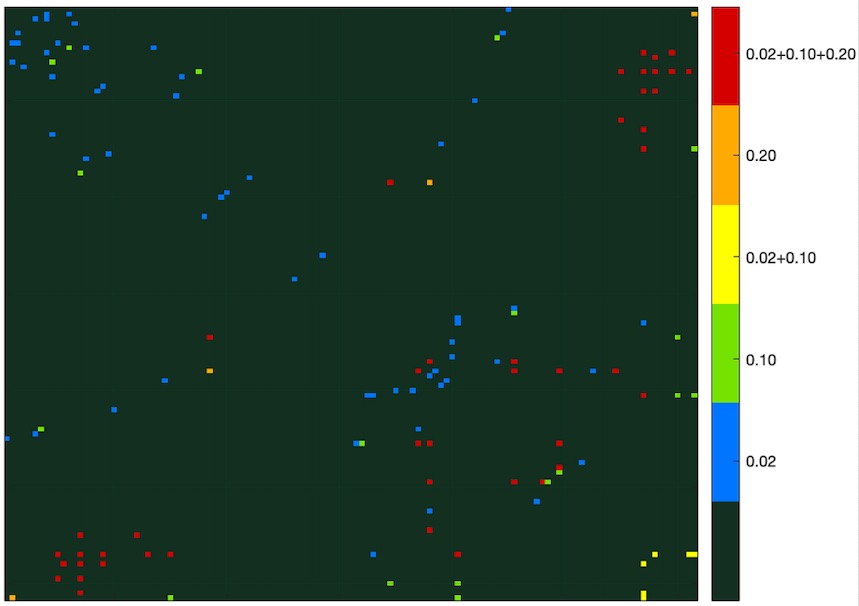


**Supplementary Figure 5.** The proportion matrix corresponds to the DTI structural disconnectivity. The color scales represent the ratio of the number of fibers in the disconnectivity matrix with WMH frequency threshold 0.2 to the number of full connectivity in each age group of 50’s.


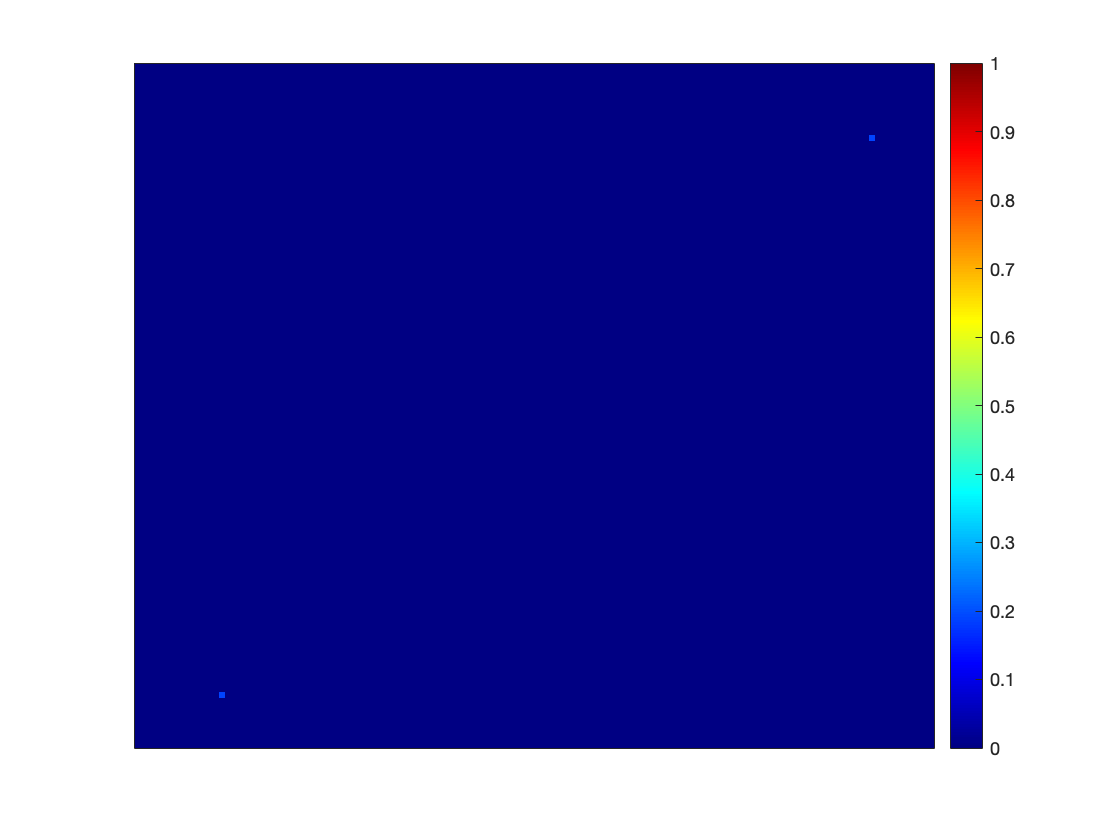


**Supplementary Figure 6.** The proportion matrix corresponding to the DTI structural disconnectivity. The color scales represent the ratio of the number of fibers in disconnectivity matrix with WMH frequency threshold 0.2 to the number of full connectivity in each age group of 60’s.


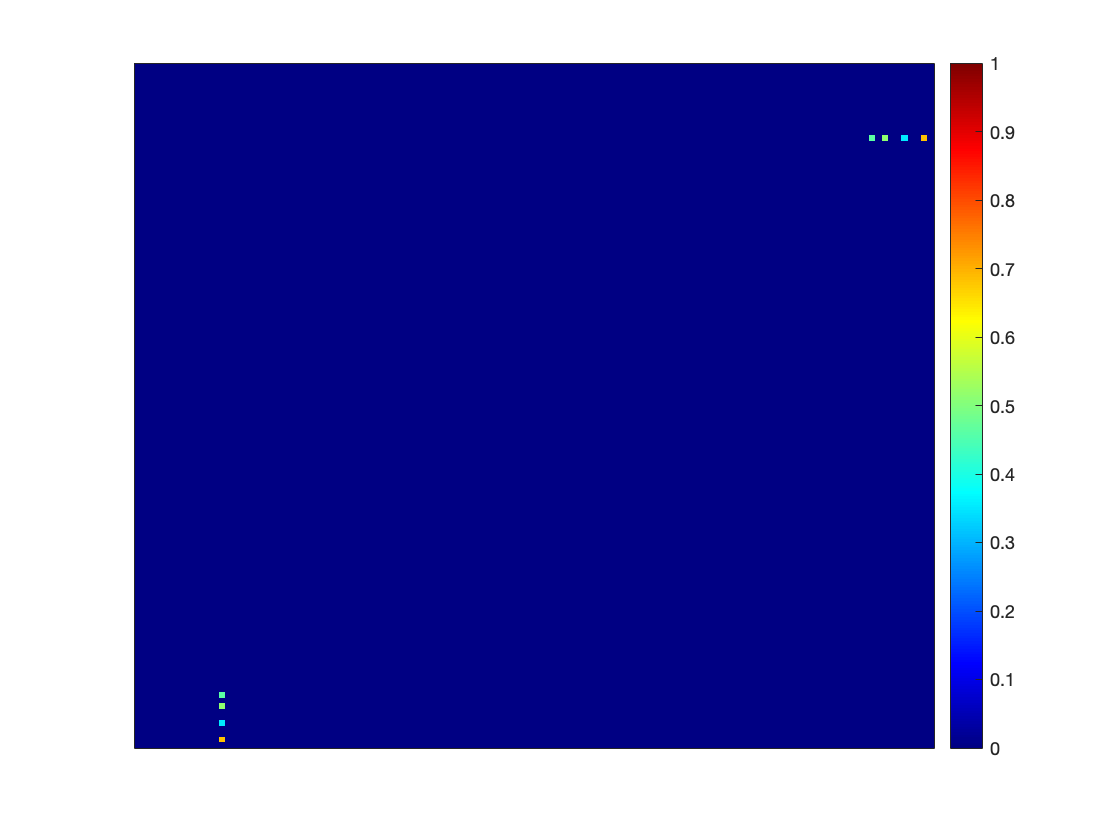


**Supplementary Figure 7.** The proportion matrix corresponds to the DTI structural disconnectivity. The color scales represent the ratio of the number of fibers in the disconnectivity matrix with WMH frequency threshold 0.2 to the number of full connectivity in each age group of the 70’s.


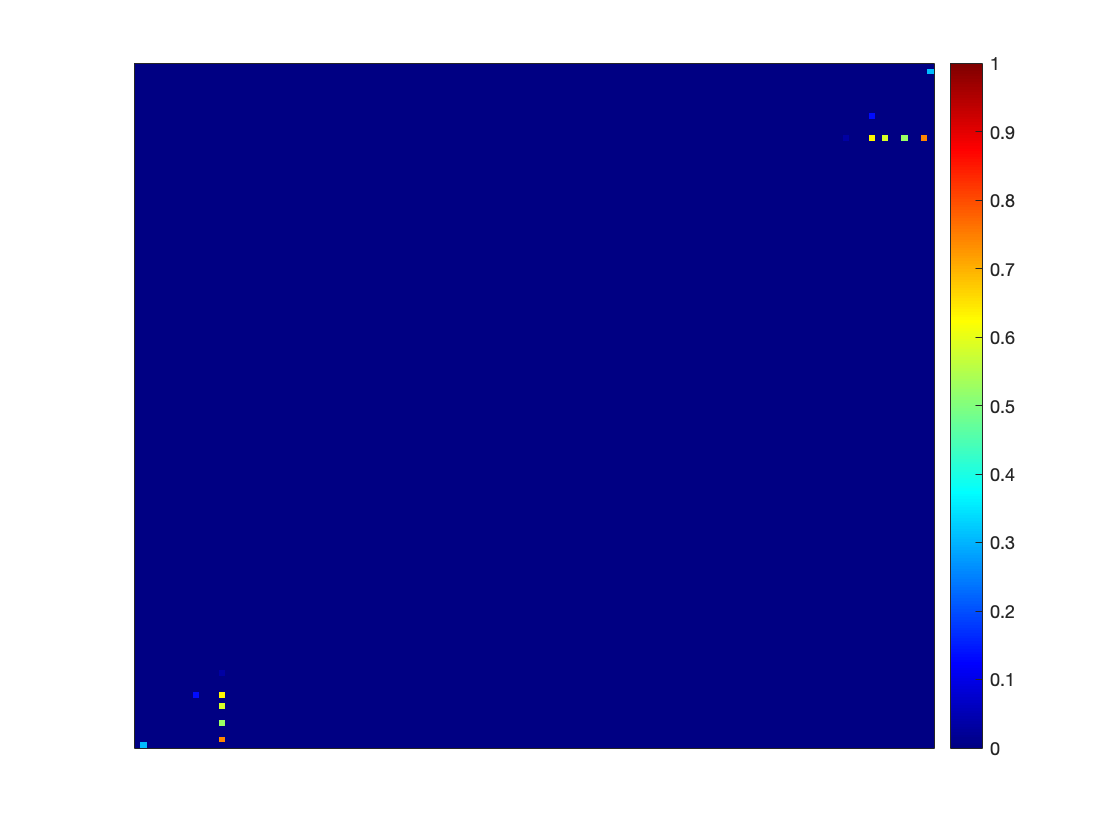


**Supplementary Figure 8.** The proportion matrix corresponds to the DTI structural disconnectivity. The color scales represent the ratio of the number of fibers in the disconnectivity matrix with WMH frequency threshold 0.2 to the number of full connectivity in each age group of 80’s.


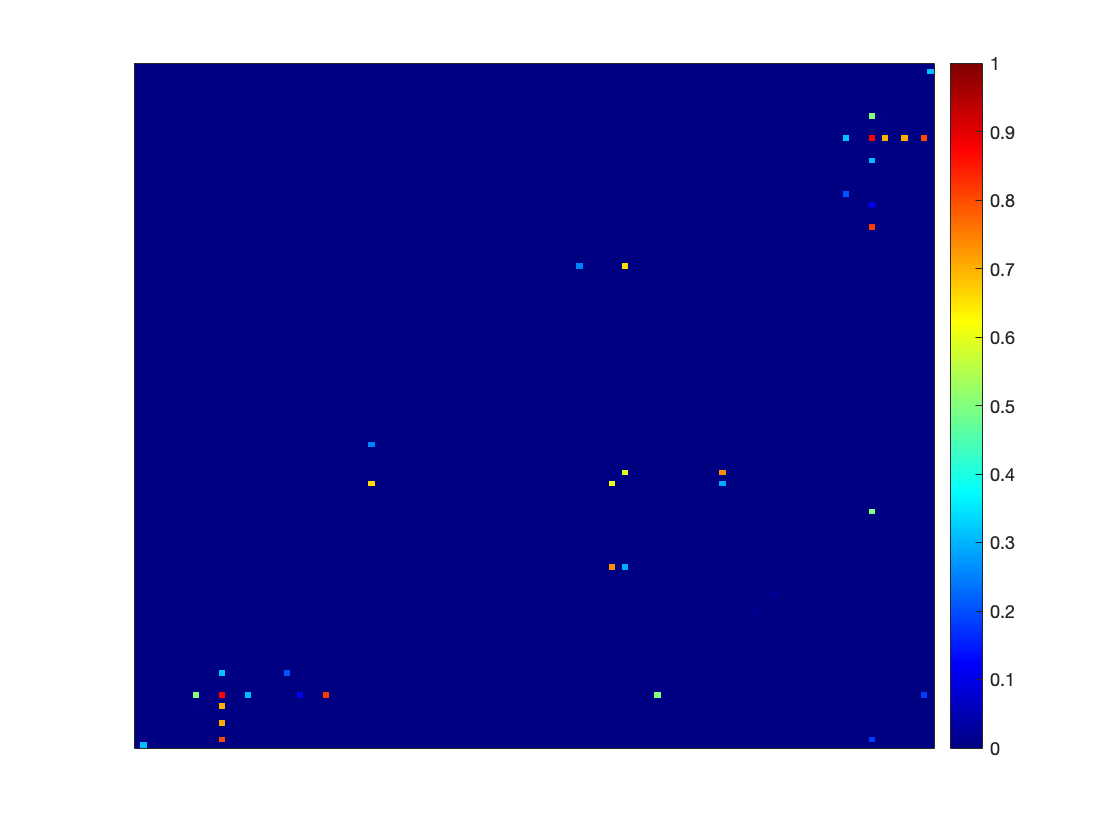

Supplement: Supplementary file 1 [file Data_Sheet_1.docx]
